# Metabolic Imaging to Assess Treatment Response to Cytotoxic and Cytostatic Agents

**DOI:** 10.3389/fonc.2016.00152

**Published:** 2016-07-15

**Authors:** Natalie J. Serkova, S. Gail Eckhardt

**Affiliations:** ^1^Department of Anesthesiology, University of Colorado Comprehensive Cancer Center, Aurora, CO, USA; ^2^Developmental Therapeutics Program, University of Colorado Comprehensive Cancer Center, Aurora, CO, USA; ^3^Division of Medical Oncology, Anschutz Medical Center, University of Colorado Denver, Aurora, CO, USA

**Keywords:** chemotherapeutics, signal transduction inhibitors, magnetic resonance spectroscopy, positron emission tomography, RECIST

## Abstract

For several decades, cytotoxic chemotherapeutic agents were considered the basis of anticancer treatment for patients with metastatic tumors. A decrease in tumor burden, assessed by volumetric computed tomography and magnetic resonance imaging, according to the response evaluation criteria in solid tumors (RECIST), was considered as a radiological response to cytotoxic chemotherapies. In addition to RECIST-based dimensional measurements, a metabolic response to cytotoxic drugs can be assessed by positron emission tomography (PET) using ^18^F-fluoro-thymidine (FLT) as a radioactive tracer for drug-disrupted DNA synthesis. The decreased ^18^FLT-PET uptake is often seen concurrently with increased apparent diffusion coefficients by diffusion-weighted imaging due to chemotherapy-induced changes in tumor cellularity. Recently, the discovery of molecular origins of tumorogenesis led to the introduction of novel signal transduction inhibitors (STIs). STIs are targeted cytostatic agents; their effect is based on a specific biological inhibition with no immediate cell death. As such, tumor size is not anymore a sensitive end point for a treatment response to STIs; novel physiological imaging end points are desirable. For receptor tyrosine kinase inhibitors as well as modulators of the downstream signaling pathways, an almost immediate inhibition in glycolytic activity (the Warburg effect) and phospholipid turnover (the Kennedy pathway) has been seen by metabolic imaging in the first 24 h of treatment. The quantitative imaging end points by magnetic resonance spectroscopy and metabolic PET (including 18F-fluoro-deoxy-glucose, FDG, and total choline) provide an early treatment response to targeted STIs, before a reduction in tumor burden can be seen.

## Introduction

The field of medical oncology has emerged in the 1950s when various chemotherapeutic drugs were used to control cancer cell growth by interfering with the cell cycle and DNA replication. Later, in the 1960s and 1970s, drugs were combined to combat the cancer at different points of the cell cycle. For several decades, cytotoxic chemotherapeutic agents were considered the basis of anticancer treatment for patients with solid tumors and metastatic (systemic) disease. A decrease in tumor burden (tumor size and metastasis size/numbers), assessed by dimensional/volumetric magnetic resonance imaging (MRI) or computed tomography (CT), was considered as a radiological response to a cytotoxic treatment regimen (1, 2).

**Figure 1:**
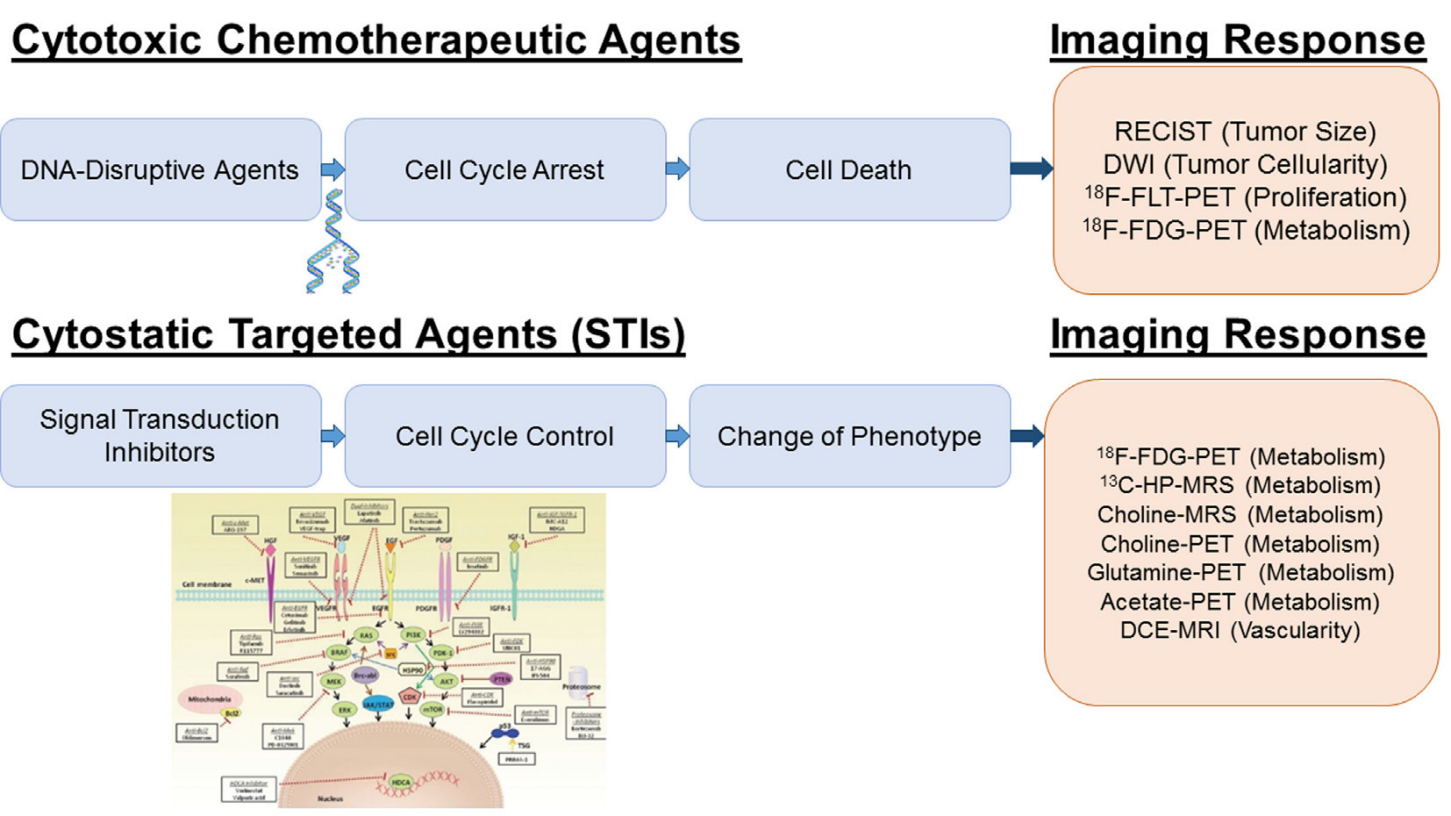
**Imaging platforms for treatment response to cytotoxic and cytostatic agents**. For DNA disrupting agents (“Cytotoxic Chemotherapeutic Agents”), increased ADC values by DWI and decreased FLT and FDG uptake by PET reflect a cytotoxic treatment response due to the decreased in tumor cellularity, DNA synthesis and metabolism. For receptor tyrosine kinase inhibitors and PI3K/AKT/mTOR inhibition (“STIs”), a specific early decrease in glycolytic activity has been reported; therefore, glucose imaging using hyperpolarized ^13^C-pyruvate MRSI or FDG-PET is most sensitive. Inhibition of the Kennedy pathway as monitored by decreased total choline MRSI or ^11^C-/^18^F-choline PET is a putative marker for the treatment response of Ras/Raf/MEK/MAPK inhibitors. Glutamine and acetate imaging can be useful for c-myc and FASN inhibitors, respectively. For antiangiogenic agents (VEGF/VEGFR2 inhibitors), DCE-MRI is the technique of choice to assess decreased perfusion and vascularity. The picture was partially adapted from Munagala et al. (7).

Recently, the discovery of molecular origins of tumorogenesis led to the introduction of novel targeted agents, the so-called signal transduction inhibitors (STIs), and their translation into the clinic (3–5). By focusing on molecular abnormalities, which are specific to the cancer cell, targeted cancer therapies have a potential to be more effective against cancer and less harmful to normal cells than “standard” chemotherapeutics. STIs are considered a cytostatic (rather than cytotoxic) treatment alternative based on a specific biological inhibition (rather than immediate cell death) (Figure 1). As such, tumor size is not a sensitive end point for the treatment response to STIs; novel physiological imaging end points are desirable (6).

Anticancer therapies are currently undergoing enormous changes. Unfortunately, this biological revolution in cancer treatment comes at a great expense; the aggregate cost of cancer care rose 60% since 2003 (8, 9). In 2014, the price for each new approved cancer drug exceeded $120,000/year of use. Therefore, the National Cancer Institute (NCI) has currently acknowledged that “there is a tremendous need to incentivize development of validated and accepted diagnostics in order to keep pace with the explosion of new, targeted drugs that are in the pipeline” (10). Advances in oncologic imaging pave the way for rapid optimization of personalized anticancer therapies through the non-invasive assessment of the mechanism of actions, efficacy and resistance development that improve clinical decision making for novel targeted agents beyond the traditional endpoints of morbidity and mortality. Among other radiological platforms, metabolic imaging – based on positron emission tomography (PET) and magnetic resonance spectroscopy (MRS) – is particularly suited for monitoring the treatment response to cytostatic STIs since the signal transduction pathways are directly linked to the aberrant metabolic phenotype exhibited in human malignancies (11–16). Introduced in 1977, ^18^F-fluoro-deoxy-glucose (FDG)-PET remains the main metabolic imaging technique for the non-invasive assessment of glucose consumption and the Warburg effect (17, 18). The use of PET has been expanded by the introduction of other radiolabeled ligands, such as amino acids and nucleosides. While tracer uptake studies represent the main strength of metabolic PET, ^1^H-MRS provides complementary metabolic information on major endogenous metabolites (19–21). In the past 10 years, advances in hyperpolarized ^13^C-MRS allowed for non-invasive assessment of metabolic activities in glucose, lipid, and amino acid metabolism in tumor-bearing animals and humans (22, 23).

## Anticancer Treatment Strategies

### Cytotoxic Drugs

Herbal and other preparations have been used for cancer treatment already in the Ancient World. The very first attempt to treat leukemia with a chemical agent (potassium arsenite) took place in 1865 by Heinrich Lissauer. Then, a treatment benefit of estrogen in prostate cancer was shown in the early 1940s. Shortly after, nitrogen mustard (mustine), now considered as truly the first chemotherapeutic agent, was discovered and applied for the treatment of lymphomas and other solid tumors. Later, with the elucidation of the double-helical structure of DNA in 1953, it was shown that nitrogen mustard chemically reacts with DNA (24). This discovery had revolutionized the treatment of various cancers and resulted in a rapid development of several cytotoxic chemotherapeutics, which affect the integrity of the cell’s genetic material (25). As such, most of classic chemotherapeutic drugs act in a cytotoxic manner, kill cells that divide rapidly, which includes *cancer cells*, immune cells, gastrointestinal (GI) tract, hair follicles, and result in a wide range of serious side effects to normal cells with high replication rate, including myelosuppression, GI toxicity, and alopecia (26–28).

Most of the cytotoxic chemotherapeutic drugs affect DNA synthesis or cell division and are commonly divided into four major classes (29–33): (i) mitotic poisons (preventing microtubule functions), (ii) DNA-reacting drugs (chemically modifying DNA as alkalyting agents), (iii) inhibitors of DNA replications (acting as antimetabolites for pyrine, pyrimidine, and thymine synthesis), and (iv) agents that change DNA topology (topoisomerase inhibitors and cytotoxic antibiotics) (Table 1).

**Table 1:** **Major classes of cytotoxic agents**.

Cytotoxic chemotherapeutics			

Mitotic poisons	DNA-reactive drugs	Inhibitors of DNA replication	Modulators of DNA topology
Vincristine (1960) Vinblastine (1960) Paclitaxel (1990) Docetaxel (1995)	N_2_-Mustard (1950) Cyclophosphamide (1960) Melphalan (1965) Mitomycin (1970) Bleomycin (1975) Cisplatin (1980) Carboplatin (1985)	Methotrexate (1955) 5-Fluorouracil (1960) Gemcitabine (1995)	Doxorubicin (1975) Amsacrine (1985) Topotecan (1995) Irinotecan (2000)

### Cytostatic Targeted Agents

The discovery of molecular targets has enabled the development of new and potentially more effective treatments for metastatic disease with considerably low toxic side effects (28). Due to our improved understanding of cancer biology and specific onco-pathways that lead to uncontrolled cell proliferation, the main focus of anticancer treatment strategies has shifted from cytotoxic chemotherapies (which lead to cell death) to cytostatic targeted STIs. This has resulted in new requirements for pharmacodynamic markers (including imaging-based end points) for therapy response and resistance development to STIs (34). Oncologic imaging represents an ideal technology to answer these questions non-invasively and in real time (35–37).

Most of the targeted agents interfere with proteins that are involved in signal transduction processes. Progressive disease, the process of tumor growth, angiogenesis, invasion, and metastasis, is largely regulated by circulating growth factors and their binding to receptor tyrosine kinases (38, 39). Inhibition of these signaling pathways as a therapeutic approach has gained a lot of attention and current strategies include: antigrowth factor antibodies, receptor antagonists, anti-receptor monoclonal antibodies, and small-molecule tyrosine kinase inhibitors (24, 40). The use of molecularly targeted anticancer drugs began with the introduction of trastuzumab and imatinib, which target HER2/neu [*human epidermal growth factor (EGF) receptor 2*] and BCR-ABL (*from Philadelphia chromosome*)/PDGFR (*platelet-derived growth factor receptor*)/c-Kit (*stem cell growth factor receptor*), for the treatment of breast cancer and chronic myeloid leukemia, respectively (41–43). Some of the signal transduction pathways commonly altered in the malignant phenotype include various the upstream receptor tyrosine kinases, such as vascular endothelial growth factor (VEGF), EGF, insulin-like growth factor (IGF1), and PDGF, as well as downstream signaling kinases, specifically, PI3K/AKT/mTOR and Ras/Raf/MEK/MAPK pathways (38, 44–48) (Table 2).

**Table 2:** **Major classes of cytostatic agents**.

Cytostatic signal transduction inhibitors					

Receptor tyrosine kinase inhibitors	PI3K/AKT/mTOR inhibitors	Ras/Raf/MEK/MAPK inhibitors	Antiangiogenic (VEGF/VEGFR2)	Hormone therapy (estrogen/androgen)	Immune checkpoint inhibitors
– Imatinib (PDGFR) – Trastuzumab (Her2) – Lapatinib (Her2) – Pertuzumab (Her2) – Gefitinib (EGFR) – Erlotinib (EGFR) – Cetuximab (EGFR) – Panitumumab (EGFR) – Picropodophyllin (IGF-1R) – Linsitinib (IGF-1R) – Pazopanib (multi)	– Everolimus (mTOR) – Temsirolimus (mTOR) – Enzastaurin (PI3K) – Afuresertib (AKT)	– Sorafenib (Raf) – Dabrafenib (BRAF) – Trametinib (MEK) – Selumetinib (MEK) – Binimetinib (MEK)	– Bevacizumab (VEGF) – Axitinib (VEGFR2)	– Estrogen receptor – Tamoxifen – Toremifene – Fulvestrant – Androgen receptor – Milutamide – Finasteride	Nivolumab (anti-PD-1) Pembrolizumab (anti-PD-1) Pidilizumab (anti-PD-1) MPDL3280A (anti-PD-L1) BMS-936559 (anti-PD-L1) MEDI4736 (anti-PD-L1)

## Imaging Treatment Response

### Response Evaluation Criteria in Solid Tumors

In the past, anatomical imaging using plain radiographs, CT, MRI, and ultrasound (US) has been applied to assess the efficacy of cytotoxic chemotherapeutics based on lesion numbers and tumor size. Response evaluation criteria in solid tumors (RECIST) to measure lesion diameters have been the “gold standard” end point for cytotoxic agents for decades (49–51). Once target lesions are measured using single linear summation (lesion diameter by RECIST) or the bilinear volumetric approach [World Health Organization (WHO)], the treatment response is usually assigned as complete response (CR), partial response (PR, >30% linear decrease), stable disease (SD), or progressive disease (PD, >20% linear increase) (52). Since the introduction of the most recent version (RECIST 1.1) in 2009, several weakness areas have been identified, including the absence of potential early indicators of response, such as functional imaging, the scarceness of validation in rare tumors, and the lack of validation for novel targeted agents. As such, attempts to optimize the RECIST criteria are still needed to accurately evaluate tumor responses.

### Advanced Imaging of Cytotoxic Response

Introduction of diffusion-weighted imaging (DWI) to assess tumor cellularity was the next step in bringing imaging endpoints from a simple volumetric measurement to a functional assessment of therapy response. Tissues with high cellularity have restricted water diffusion, which can be quantitatively assessed by calculation of water apparent diffusion coefficients (ADC) from DWI, which are considerably low in fast proliferating tumors (53–56). Aggressive tumor and metastatic lesions are repeatedly reported to have ADC values below 1.2 × 10^−3^ mm^2^/s. A decrease in tumor cellularity and induction of cell death by cytotoxic chemotherapeutics results in increased ADC values, and increased ADC values have been reported as imaging biomarkers for chemotherapy response (57–60). For example, in breast cancer patients, an increase in ADC values in responders (as early as one cycle of neoadjuvant chemotherapy) is a good predictor for the later decrease in MRI tumor diameters (59).

Alternative imaging platforms for cytotoxic response are based on metabolic PET. Malignant tissues are chiefly composed of rapidly dividing cells, which exhibit highly upregulated DNA synthesis. ^18^F-fluoro-3-deoxy-thymidine (^18^FLT) is a PET tracer for tumor cell proliferation (based on the high thymidine uptake by proliferating cells in the pyrimidine salvage pathway during S-phase). Although not highly specific (61, 62), a decreased signal intensity in ^18^FLT-PET can be observed when DNA synthesis is disrupted by chemotherapeutic agents, often simultaneously with a profound DWI response by MRI (63–65). Another PET application is based on the fact that cancer cells use large amounts of glucose as a direct source of energy to permit the exaggerated utilization of amino acids and nucleosides in the synthesis of DNA. The radioactive glucose analog FDG is the most widely used tracer in oncologic PET/CT to assess metabolic cancer aggressiveness based on high glucose uptake and metabolism through high GLUT-1 transporters and hexokinase expression/activity (66). It has been shown that in patients with lung, breast, head-and-neck, esophageal, colorectal cancers, and lymphoma, the standardized uptake values of FDG decrease in responding tumors after one cycle of chemotherapy (18, 67).

### Imaging in Radiation Oncology

Radiation therapy is used as part of cancer treatment, mostly in combination with systemic chemotherapy, in roughly 50% of all cancer cases. It is especially effective in head-and-neck, breast, prostate, cervical, and skin cancer, while colorectal cancer, soft tissue sarcomas, and high-grade gliomas usually show only a limited response rate. The posttreatment effects of radiotherapy are attributed to tumor inflammation, cell necrosis and often increased angiogenesis (68, 69). Clinically, FDG-PET/CT is frequently acquired at the baseline for radiation treatment planning since high metabolic activity is regarded as a positive predictive factor for treatment response (70). A profound metabolic response, as detected by decreased FDG uptake values on postradiation PET/CT scans, has correlated with high progression-free survival rates in almost all types of cancer (71–73). Hyperpolarized MRS using [1-^13^C]-pyruvate also showed a significant decrease in lactate production as early as 96 h after irradiation in orthotopic rat glioma models (74) and colorectal flank xenografts (75).

### Metabolic Imaging of Signal Transduction Inhibition

Changes in tumor size, the “gold-standard” of tumor response for cytotoxic chemotherapeutic agents, are often not useful in monitoring therapy response in the first cycles of STI-based therapy. Humanized monoclonal antibodies and small-molecule receptor tyrosine kinase inhibitors have been developed to target epidermal growth factor receptors (EGFR), platelet-derived growth factor receptor (PDGFR), and insulin-like growth factor receptor (IGF-1R), which are overexpressed in a significant number of human malignancies. These inhibitors of the receptor activity include gefitinib, erlotinib, imatinib, cetuximab, and trastuzumab and have the most profound metabolic effects by inhibiting both glucose and choline metabolism, which are two main metabolic hallmarks of cancer (76–78). Therefore, the imaging response to receptor inhibitors has been successfully monitored – both preclinically and clinically – using glucose-based (FDG-PET and hyperpolarized ^13^C-MRS) (79–89) and choline-based (^1^H-/^31^P-MRS and choline-PET) metabolic imaging (90–92). The metabolic response on FDG-PET was seen as early as 8 days after initiation of treatment (93).

Upstream receptor upregulation leads to the downstream activation of two main intracellular onco-pathways: the GTPase Ras/Raf/MEK/MAPK and the lipid kinase PI3K/AKT/mTOR pathways. It has been convincingly shown that the PI3K/AKT/mTOR pathway directly downregulates glucose metabolism: a significant decrease in glucose uptake, lactate production, and glycolytic enzyme expression has been seen with several mTOR (94–97) and PI3K inhibitors (98, 99). ^13^C-MRSI measurements of the conversion of hyperpolarized [1-^13^C]-pyruvate into lactate have been used to image the decrease in tumor LDH activity due to the inhibition of the PI3K/AKT/mTOR pathway. Confirming these MRS data, the decreased FDG uptake was seen on PET scans upon mTOR/PI3K inhibition (97, 100, 101). Most recently, the US Centers for Medicare and Medicaid Services (CMS) have approved the coverages of FDG-PET/CT for treatment response in most solid tumors, especially for the treatment strategies based on receptor tyrosine kinase inhibitors and PI3K/AKT/mTOR mediated pathways (88). FDG-PET is intrinsically a quantitative imaging technique for early STI treatment response based on calculations of the standardized uptake value (SUV) of FDG uptake (77, 97). An improved quantification of treatment response based on decreased SUVs has been introduced as the PET response criteria in solid tumors (PERCIST 1.0) (102).

In contrast, MEK inhibitors, with MEK being the main therapeutic target from the Ras/Raf/MEK/MAPK pathway, do not exhibit a considerable glycolytic effect as revealed by FDG-PET and hyperpolarized MRS (64, 103, 104), but significantly reduce choline metabolism (104–106). Choline is a precursor of phosphatidylcholine, the major cell membrane phospholipid. Ras/Raf/MEK/MAPK pathway inhibition leads to the decrease in choline transporters and might also influence the activity of choline kinase (CHKα) leading to a significant decrease of the total choline peak detected by MRS. ^11^C- or ^18^F-choline PET/CT can be used to detect a significant decrease in tracer uptake following treatment with various targeted STIs, especially those from the Ras/Raf/MEK/MAPK pathway (107).

While the PI3K/AKT/mTOR pathway is considered to be “glucose-dependent,” recent studies have shown that the MYC oncogene, which encodes a master transcription factor c-Myc, regulates glutamine catabolism to fuel growth and proliferation of cancer cells through upregulating glutaminase (GLS) (108–110). The first success in imaging glutaminase activity by MRS was achieved using hyperpolarized ^13^C-glutamine (111). Recently, ^11^C- and ^18^F-labeled glutamine has been synthesized and successfully utilized for non-invasive PET detection of c-Myc tumors in rodent models (112, 113). In addition, recent *in vitro* MRS studies with c-Myc overexpressed breast cancer cells showed a significant suppression of glutaminolysis when treated with aminooxyacetate, an inhibitor of aminotransferases involved in amino acid metabolism (114, 115). Several c-Myc inhibitors are now in preclinical testing, and glutamine-PET will be an obvious technique of choice for monitoring metabolic treatment response.

Positron emission tomography measurements of the uptake and trapping of ^11^C-acetate, due to the increased expression of fatty acid synthase (FASN), have been used to detect prostate cancer and hepatocellular carcinoma – two cancers where FDG-PET evaluations have proven to be challenging or non-effective (116–118). The use of ^11^C-acetate PET/CT can be useful while assessing treatment response to FASN and fat oxidation inhibitors, such as orlistat and etomoxir, in prostate cancer (119, 120).

Finally, the therapeutic efficacy of antiangiogenic agents targeting the VEGF/VEGFR2 pathway can be monitored using dynamic contrast-enhanced (DCE)-MRI (121–123). The time-dependent signal enhancement on dynamic T1-weighted MRI reflects intratumoral contrast delivery after an intravenous injection of gadolinium contrast and is proportional to tumor perfusion and vascularity. A dramatic decrease in T1-enhancement, calculated as decreased gadolinium transfer constant, *K*^trans^, or the decreased area under the enhancement curve, AUC, was seen after tumor treatment with VEGF antibodies, such as bevasizumab, or VEGFR2 tyrosine kinase inhibitors.

### Imaging of Hormone- and Immune-Based Therapies

In addition to cytotoxic DNA-interfering agents and cytostatic STIs, other classes of anticancer drugs have been developed. The most promising are hormones and hormone antagonists for breast, prostate, and endocrine tumors. ^18^F-labeled PET tracers for androgen and estrogen receptor imaging have been developed and tested in animal models (124, 125); ^18^F-fluoro-estradiol (FES) is undergoing clinical trials to monitor early treatment response to aromatase inhibitors, such as tamoxifen and fulvestrant, in ER+ breast cancer patients (126, 127). Finally, the most exciting area in anticancer treatment lies in cancer immunotherapy and novel immunomodulatory targeted agents (128). The inhibitors of the programed cell death receptor PD-1 and its ligands PDL-1, such as nivolumab and pembrolizumab, have recently shown a promising antitumor activity in melanoma and lung cancers and, to some degree, in triple-negative breast cancers (129–131). The most recent report from the phase Ib on pembrolizumab in patients with advanced melanoma clearly demonstrated that conventional RECIST criteria are not appropriate for the adequate assessment of immune response and might underestimate the benefit of the immune checkpoint blockade in 15% of treated patients leading to premature cessation of treatment (132). However, the metabolic aspects of this activated antitumor immune response are still to be elucidated.

## Conclusion

For “classic” chemotherapeutic agents, increased ADC values by DWI reflect an early cytotoxic treatment response due to decreased tumor cellularity and are an attractive alternative to volumetric imaging. For novel STIs, physiological and metabolic imaging protocols should be carefully chosen based on a particular signal transduction pathway involved. For receptor tyrosine kinase inhibitors and PI3K/AKT/mTOR inhibition, a specific decrease in glycolytic activity has been reported; therefore, glucose imaging using hyperpolarized ^13^C-pyruvate MRSI or FDG-PET is most sensitive. Inhibition of the Kennedy pathway as monitored by decreased total choline MRSI or ^11^C-/^18^F-choline PET is a putative marker for the treatment response of Ras/Raf/MEK/MAPK inhibitors. For antiangiogenic agents (VEGF/VEGFR2 inhibitors), DCE-MRI is the technique of choice to assess decreased perfusion and vascularity.

Introduction of novel targeted STIs, including immune checkpoint inhibitors, requires a robust validation of novel quantitative imaging end points from PET, MRS, and other supporting imaging platforms that characterize early physiological and metabolic treatment response before a reduction in tumor burden can be seen (6). Using medical imaging to distinguish responders versus non-responders at early time points can contribute to improved tailoring of therapy in individual cancer patients. The new term, *radiogenomics*, has recently been introduced to link quantitative physiological imaging end points with molecular markers of signal transduction pathway inhibition (133).

## Author Contributions

Both the authors listed, have made substantial, direct, and intel-lectual contribution to the work, and approved it for publication.

## Conflict of Interest Statement

The authors declare that the research was conducted in the absence of any commercial or financial relationships that could be construed as a potential conflict of interest. The reviewers M-FP, KG, and BK and handling Editor declared their shared affiliation, and the handling Editor/Specialty Chief Editor states that the process nevertheless met the standards of a fair and objective review.
